# A methodological framework to assess the carbon balance of tropical managed forests

**DOI:** 10.1186/s13021-016-0056-7

**Published:** 2016-07-29

**Authors:** Camille Piponiot, Antoine Cabon, Laurent Descroix, Aurélie Dourdain, Lucas Mazzei, Benjamin Ouliac, Ervan Rutishauser, Plinio Sist, Bruno Hérault

**Affiliations:** 1 Université de la Guyane, UMR EcoFoG (AgroParisTech, CNRS, Inra, Université des Antilles, Cirad), Campus agronomique, 97310 Kourou, French Guiana; 2 Centre Tecnològic Forestal de Catalunya, Crta. de Sant Llorenç de Morunys, Km.2, 25280 Solsona, Spain; 3 ONF-Guyane, Réserve de Montabo, 97307 Cayenne, French Guiana; 4 Cirad, UMR EcoFoG (AgroParisTech, CNRS, Inra, Université de la Guyane, Université des Antilles), Campus agronomique, 97310 Kourou, French Guiana; 5 EMBRAPA Amazônia Oriental, Trav. Dr. Enéas Pinheiro, Belém, Brazil; 6 Guyane Energie Climat, 16 rue Victor Schoelcher, 97300 Cayenne, French Guiana; 7 CarboForExpert, 1248 Hermance, Switzerland; 8 Cirad UR Forêts et Sociétés, 34398 Montpellier, France; 9 CNRS, UMR EcoFoG (AgroParisTech, Inra, Université de la Guyane, Université des Antilles, Cirad), Campus Agronomique, 97310 Kourou, French Guiana

**Keywords:** Carbon cycle, Selective logging, Error propagation, Amazonia, Production forests

## Abstract

**Background:**

Managed forests are a major component of tropical landscapes. Production forests as designated by national forest services cover up to 400 million ha, i.e. half of the forested area in the humid tropics. Forest management thus plays a major role in the global carbon budget, but with a lack of unified method to estimate carbon fluxes from tropical managed forests. In this study we propose a new time- and spatially-explicit methodology to estimate the above-ground carbon budget of selective logging at regional scale.

**Results:**

The yearly balance of a logging unit, i.e. the elementary management unit of a forest estate, is modelled by aggregating three sub-models encompassing (i) emissions from extracted wood, (ii) emissions from logging damage and deforested areas and (iii) carbon storage from post-logging recovery. Models are parametrised and uncertainties are propagated through a MCMC algorithm. As a case study, we used 38 years of National Forest Inventories in French Guiana, northeastern Amazonia, to estimate the above-ground carbon balance (i.e. the net carbon exchange with the atmosphere) of selectively logged forests. Over this period, the net carbon balance of selective logging in the French Guianan Permanent Forest Estate is estimated to be comprised between 0.12 and 1.33 Tg C, with a median value of 0.64 Tg C. Uncertainties over the model could be diminished by improving the accuracy of both logging damage and large woody necromass decay submodels.

**Conclusions:**

We propose an innovating carbon accounting framework relying upon basic logging statistics. This flexible tool allows carbon budget of tropical managed forests to be estimated in a wide range of tropical regions.

## Background

There is a growing interest of the international community in tropical forests and their key role in the global carbon cycle. In particular, tropical managed forests, i.e. tropical forests managed for timber production, are important in the context of climate change mitigation as they might either act as carbon source or sink, depending on their management type and cutting cycle length [1]. With half of tropical humid forests, i.e. more than 400 million ha, designated by National Forest Services (NFS) as production forests [2], forest management will play a decisive role in future global carbon cycle. However, while there is extensive scientific literature on undisturbed tropical forests, our knowledge on carbon balance (i.e. the net carbon exchange with the atmosphere) in managed forests remains to be improved. With international programs like REDD+ (Reducing Emissions from Deforestation and Degradation) aiming at mitigating carbon emissions, notably through improved forest management, it is essential to have a clear understanding of the carbon balance of tropical managed forests in order to develop unified accounting methods for carbon emissions.

The second “D” of REDD+, forest degradation, refers to the reduction of carbon stocks in areas that have retained sufficient canopy cover to be still considered as forests [3]. In tropical forests, selective logging is generally considered an important factor of forest degradation. Selective logging consists in harvesting only a few trees and leaving the rest of the forest to natural regeneration until the next logging event. Selective logging practices result in long-lasting carbon emissions to the atmosphere [4]. In the sole Brazilian Amazon, forestry-related carbon emissions are estimated to be equivalent to 60–123 % of deforestation emissions [5]. Emissions come from (i) the extracted wood, (ii) logging damage, i.e. trees killed (purposely or incidentally) during logging operations and (iii) skid trails, logging roads and decks used for log yarding. Logging operations (e.g. tree felling, log yarding or skidding) induce incidental damage to surrounding trees, proportional to logging intensity in conventional [6] or reduced impact logging [7]. Injured or smashed trees generally die soon after logging operation [8, 9], and, along with log residuals such as crowns or stumps, are left over in the forest. The quite slow decay of these woody debris and harvested logs is a long-term source of carbon to the atmosphere.

However selective logging does not induce carbon emissions only. Logging gaps and incidental mortality release local competition for key resources, such as light [10], and enhance carbon accumulation in remnant trees [11]. Some local studies suggest that carbon emissions peak soon after logging (5–10 years), while tree growth and recruitment counterbalance tree mortality after a decade, resulting in positive carbon accumulation in logged forests until an equilibrium is reached [12]. If not further disturbed, forests recover most of their initial carbon stock at the end of the cutting cycle, depending on logging intensity and damage [13, 14]. While skid trails and logging decks generally close rapidly after logging, logging roads may be reopened and persist for decades in the landscape [15].

Most studies on the carbon balance of selectively logged forests have estimated pre- and post-logging carbon stocks with remote sensing methods [16], allowing large-scale estimation of logging extent and quantification of canopy damage [14, 17, 18]. However optical remote sensing methods require further field calibration to avoid estimation biases [17, 19]. Accurate estimation of carbon loss and recovery based on evolution of canopy openness is questionable, as ground damage remain poorly assessed with remote sensing and canopy rapidly closes with crown expansion of survivors. [20]. Another problem lies in estimating carbon recovery rates when canopy gaps rapidly close with crown expansion of survivors and growth of recruits [21]. LiDAR and RADAR are a promising tools to overcome those limitations but remain expensive to be applied over large areas [22]. The International Panel on Climate Change [23] recommends basic methods based on activity data, e.g. the annual volume of extracted roundwood (in m^3^·year^−1^) or the annual logged area (in ha·year^−1^). So far, only a few studies have estimated the carbon balance of tropical logged forests from activity data. Griscom et al. [24] estimated carbon emissions at concession level with detailed maps of skidtrails, logged trees, damage and logging decks. But such maps are generally not available at regional level impeding the wide-use of this approach. Pearson et al. [25] developed emissions factors for the main logging compartments to be multiplied to extracted volumes, as a surrogate for carbon emissions from selective logging. For sake of simplicity, most studies have estimated committed emissions, i.e. all the carbon lost by logging is assumed to return instantaneously to the atmosphere. This choice is highly questionable as carbon emissions are spread over decades [4]. Some studies have indeed adopted a more dynamic approach, considering carbon emissions through time in managed forests [26]. However to date there is no method assessing all carbon fluxes (carbon accumulation as well as all emissions) from tropical managed forests through time.

In this study we developed a methodological framework to estimate the above-ground carbon fluxes from selective logging. Carbon fluxes are assessed first at the logging unit scale, i.e. the elementary management unit of the forest estate, and then integrated in space and time to estimate the regional carbon balance over time. The annual carbon net change of a given logging unit is estimated by aggregating carbon fluxes of three sub-models: (1) emissions from extracted wood, (2) emissions from woody debris (logging residues, damage, road and logging deck openings) left in the forest, and (3) sequestration from forest regrowth. Some submodels come from the literature while others were specifically developed and parameterised in a Bayesian framework for this study. Uncertainty over the global model are propagated through a Markov Chain Monte Carlo algorithm.

**Fig. 1:**
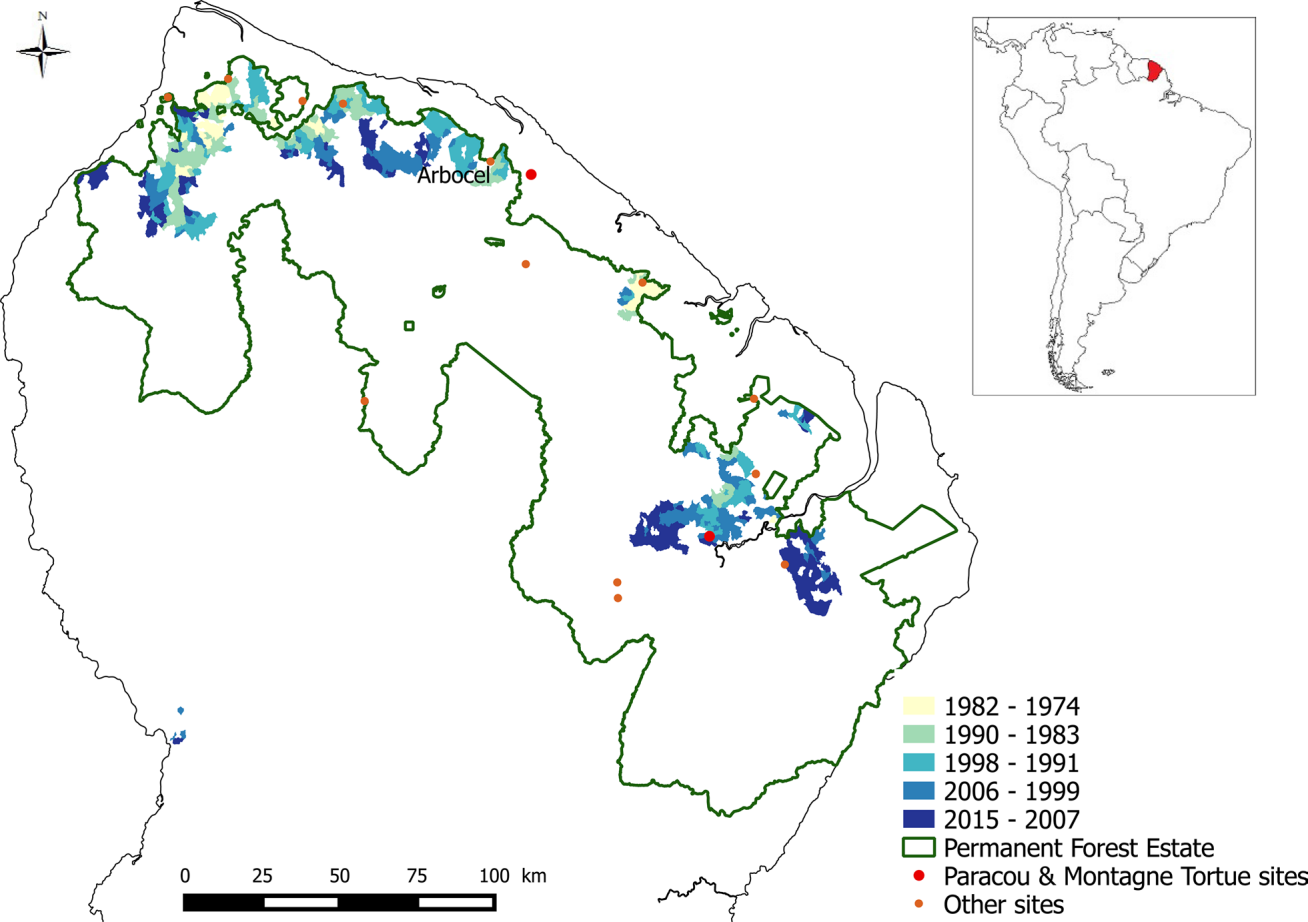
Map of the Permanent Forest Estate in French Guiana. Logging units are* coloured* according to the year of logging: from *white and light blue* for most anciently logged plots to* dark blue* for most recently logged plots.* Dots* are experimental sites from the Guyafor network:* red dots* have both logged and control plots (Paracou and Tortue sites);* smaller orange dots* have only control plots. The borders of the Permanent Forest Estate are the* bold green lines*

**Fig. 2:**
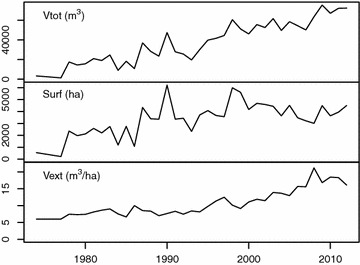
National Forest Service statistics on selective logging in the French Guiana Permanent Forest Estate between 1974–2012.* Top graph* annual extracted volumes of roundwood *Vtot* ( m^3^) ;* middle graph* annual logged areas *Surf* (ha);* bottom graph* annual logging intensities *Vext* (m^3^·ha^−1^)

Our case study is French Guiana, a French territory located in the Guiana shield (northeastern Amazonia) with a total area of 84,000 km^2^. Above 95 % of its area (8Mha) is occupied by tropical rainforest: timber extraction by selective logging is the 3rd economic sector. Selective logging occurs exclusively in the Permanent Forest Estate (2.4 Mha) (Fig. 1), an area managed by the French National Forest Service (NFS). We used 38-year NFS statistics on 1155 logging units (Fig. 2) to estimate the carbon balance of managed forests at countrywide scale.

## Methods

### The model framework

*Methods overview* We created a model to assess annual above-ground carbon fluxes at the logging unit scale, from the logging year up to the last year of NFS statistics, and then integrated these results over time and space. Conventionally, fluxes from carbon emissions are positive and fluxes from carbon accumulation are negative.

**Fig. 3:**
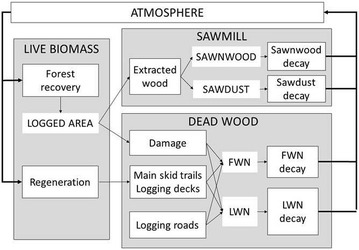
Overall scheme of the carbon balance and submodels articulation.* Thick arrows* are carbon fluxes.* Grey boxes* are the three carbon compartments: Sawmill, Dead Wood and Live Biomass. Submodels are in surrounded white boxes. FWN and LWN are fine and large woody necromass respectively

We modeled separately (1) emissions from extracted wood decay (Sawmill); (2) emissions from woody debris decay (Dead wood), divided into (2.a) logging damage and (2.b) deforestation on logging roads, main skid trails and logging decks; (3) sequestration from forest regrowth (3.a) in logging gaps and (3.b) on abandoned skid trails (Live biomass) (Fig. 3). For each submodel we first assessed the evolution of post-logging carbon stocks (in MgC·ha^−1^) in each logging unit. Annual carbon fluxes at time *t* (in MgC·year^−1^) are obtained by calculating the difference between stocks at time *t* and *t*−1, and multiplied by the logged area (in ha). Model parameters are considered as uncertain quantities, and the error is propagated with Monte Carlo methods. Doing so, we were able to track the annual carbon budget in each logging unit, and to conduct simultaneously a sensitivity analysis to identify the main sources of uncertainties in the global model.

*Calibration data* In some cases, submodels come from the literature. In other cases, and especially when there was no existing model, submodels were developed and parametrised with data from Amazonian permanent forest plots. We used data from control plots to assess pre-logging forest structure variables and logged plots to assess post-logging dynamics. Two networks of permanent forest plots were used in this study: (i) Guyafor in French Guiana (regional scale) (Fig. 1); (ii) TmFO, a network of logged plots spread across Amazonia (continental scale) [27]. From Guyafor, we used data from 11 sites, all containing control plots and two of them (Montagne Tortue, Paracou) also containing logged plots. From the TmFO network, we used data from four Amazonian sites (Jari, Tapajos, Tortue, Paracou) that have logged plots where extracted volumes have been measured. To calibrate the model of forest regeneration on logging decks and main skid trails, we used data from a 25-ha plot in French Guiana, Arbocel (see Fig. 1): the plot was clearcut in 1976 and then left to natural regeneration [28]. Some submodels and/or calibration data are specific to the context of selective logging in French Guiana: adapting this framework to other areas, countries or for upscaling purposes may require new data or new submodels to be regenerated. However, the general structure and the articulation of the different submodels is generic enough to be applied elsewhere.

*Input data* To run the model, we need basic NFS statistics available for every logging unit *p*: (i) the logged surface $Surf_p$ (ha), corresponding in French Guiana to on average 50 % of the total area, (ii) the total extracted volume $Vtot_p$ (m$^3$), (iii) the year of logging $t0_p$ and (iv) the initial above-ground carbon stock $ACS_p$ (MgC·ha$^{-1}$).

For our study, statistics on selective logging in the Permanent Forest Estate over the 1974–2012 time period were provided by the French NFS. According to NFS expertise, between 1974 and 1994 logging intensities are assumed to be equal to 6 m$^3$·ha$^{-1}$ in the western forests, and equal to 10 m$^3$·ha$^{-1}$ in the rest of the Permanent Forest Estate. After 1994, logging statistics are highly reliable and logging intensities $Vext_p$ are calculated as follows:

1 $$\begin{aligned} Vext_p = \frac{Vtot_p}{Surf_p} \end{aligned}$$

Initial above-ground carbon stocks were extracted from the recently developed carbon map of French Guiana [29].

### Sawmill

The carbon $Ext_p$ (MgC·ha$^{-1}$) of logs extracted from the logging unit *p* is defined as :

2 $$\begin{aligned} Ext_p = Vext_p \times dext \times 0.5 \end{aligned}$$

where $Vext_p$ is the logging intensity (m$^3$·ha$^{-1}$), the biomass is assumed to be 50 % carbon [30] and $dext=0.736$ is the mean wood density of timber species in French Guiana. *dext* has been estimated as the mean wood density of *Dicorynia guianensis* (*d* = 0.76), *Qualea rosea* (*d* = 0.725) and *Sextonia rubra* (*d* = 0.65) [31], weighted by their relative contribution to annual timber extraction [32].

The carbon stored in extracted wood ($Ext_p$) is then transformed into sawnwood with an efficiency of $f_{SW}=0.33$ [33], the remaining 67 % consisting of sawdust. Sawnwood and sawdust then decompose with different decay rates. Sawdust is considered to decompose entirely within the first year after logging [34]. The quantity of sawdust $DecSD_{p}$ (MgC·ha$^{-1}$) in the logging unit *p* at the year of logging $t0_p$ is:

3 $$\begin{aligned} DecSD_{p} = (1-f_{SW}) \times Ext_p \end{aligned}$$

Sawnwood decay is approximated with an exponential decay. The carbon in sawnwood $DecSW_{p,t}$ (MgC·ha$^{-1}$) from logging unit *p* at time *t* is :

4 $$\begin{aligned} DecSW_{p,t} = f_{SW} \times Ext_p \times exp \Big ( -\lambda _{SW} \times (t-t0_p) \Big ) \end{aligned}$$

with $Ext_p$ the carbon in extracted wood, $t0_p$ the year of logging and $\lambda _{SW}$ the decay rate of sawnwood in the tropics estimated as

5 $$\begin{aligned} \lambda _{SW}=\frac{ln(2)}{t_{0.5}} \end{aligned}$$

with

6 $$\begin{aligned} t_{0.5} \sim N_{t0}(30, 15^2) \end{aligned}$$

where $N_{t0}(30, 15^2)$ is the normal distribution truncated at zero with mean equal to 30 and standard deviation equal to 15, according to IPCC guidelines [30]. So far, no life cycle analysis has yet been done for woody products in the tropics and adapting the framework developed in Europe and North America in tropical countries remains challenging [35].

### Dead wood

Both logging damage and deforestation on roads, main skid trails and logging decks are sources of dead wood that is left in the forest.

#### Logging damage

**Fig. 4:**
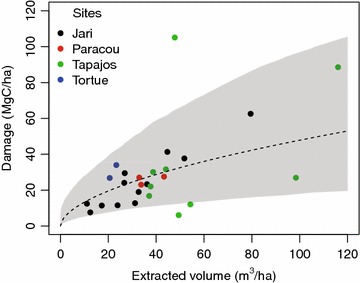
Modelling logging damage (MgC·ha$^{-1}$) from extracted wood (m$^3$·ha$^{-1}$). The* dashed line* is the prediction with maximum likelihood, the shaded area is the 95 % credibility interval on the prediction. Data are taken from four Amazonian sites: Jari (*black*), Paracou (*red*), Tortue (*green*), Tapajos (*blue*)

The logging damage $Dam_p$ (MgC·ha$^{-1}$) within the logging unit *p* consists in above-ground carbon in trees harvested or killed (purposely or incidentally) during logging operations (tree felling and log yarding or skidding) minus carbon of extracted logs ($Ext_p$). Logging damage were assessed with data from 4 TmFO sites in the Eastern Amazon, where we have logging intensities $Vext_p$ and logging damage $Dam_p$ (Fig. 4). Logging gaps and secondary skid trails are accounted for as logging damage, whereas main skid trails and logging roads are located outside of calibration plots and are considered as deforestation.

The relationship between $Vext_p$ and $Dam_p$ was modelled as follows:

7 $$\begin{aligned} Dam_{p}\sim lnN(\theta _0+\theta _1 \times Vext_p;\sigma _{dam}^2) \end{aligned}$$

where $lnN(\mu , \sigma _{dam}^2)$ is the lognormal distribution of mean $\mu$ and standard deviation $\sigma _{dam}$. We chose the lognormal distribution to have errors proportional to the extracted volume, i.e. damage is less predictable for higher logging intensities.

The parameters $(\theta _0,\theta _1, \sigma _{dam})$ were estimated with a Bayesian inference, using a Markov Chain Monte Carlo method to obtain the posterior joint distribution. Uninformative priors were chosen. Each observation (one plot) was given a weight equal to the area of the plot.

#### Deforestation

Some basic infrastructure (logging roads, main skid trails, logging decks) is generally set up to store boles and get them out of the forest. As it has to be easily reached from every part of a logging unit *p*, the total area cleared for infrastructure is assumed to be proportional to the total logged area $Surf_p$. The length of logging roads may depend on the topography but, in the French Guiana Permanent Forest Estate, the topography is quite homogeneous.  

*Logging roads* According to NFS expertise (personal communication):

8 $$\begin{aligned} \delta _{p} \sim U(0.12;0.25) \end{aligned}$$

where $\delta _{p}$ is the entire width of clearing for logging roads in logging unit *p* (expressed in hectometers hm). Based on the study of Guitet et al. [36], we fit the length of logging roads $\eta _{p}$ (hm·ha$^{-1}$) as:

9 $$\begin{aligned} \eta _{p} \sim exp(17.95) \end{aligned}$$

The carbon removed for road clearing $Road_p$ (MgC·ha$^{-1}$) and left to decay in the logging unit *p* is :

10 $$\begin{aligned} Road_p = ACS_p \times \delta _{p} \times \eta _{p} \end{aligned}$$

where $ACS_p$ is the initial above-ground carbon stock (MgC·ha$^{-1}$).

*Main skid trails* Main skid trails are built to allow the skidders to get in the logging units. According to Guitet et al. [36]:

11 $$\begin{aligned} SurfST_p \sim U(0.04;0.10) \end{aligned}$$

where $SurfST_p$ is the area (ha) of main skid trails per hectare logged. As large trees are generally avoided by skidders during yarding, we assumed that only trees with *DBH* (Diameter at Breast Height) ≤50 cm were killed. For a logging unit *p*, the above-ground carbon removed on skid trails $STrail_p$ (Mg·ha$^{-1}$) is:

12 $$\begin{aligned} STrail_p = ACS_p \times SurfST_p \times f_{50} \end{aligned}$$

where $ACS_p$ is the initial above-ground carbon stock of the logging unit *p* (Mg·ha$^{-1}$) and $f_{50}$ is the proportion of $ACS_p$ in trees* DBH* <50 cm. $f_{50}$ was estimated with data from the control plots of the 11 Guyafor sites. For one site *j*, the proportion $f_{50,j}$ was calculated as follows:

13 $$\begin{aligned} f_{50,j} = \frac{\sum \nolimits _{DBH_k \le 50} ACS_{j,k}}{\sum \nolimits _k ACS_{j,k}} \end{aligned}$$

where $\sum \nolimits _{DBH \le 50} ACS_{j,k}$ is the above-ground carbon of all trees with $DBH \le 50$ cm and $\sum \nolimits _k ACS_{j,k}$ is the total above-ground carbon of the site. We report the obtained distribution in Table 1.

**Table 1:** Parameters of the carbon balance model

Submodel	Parameter	Distribution	Justification	Data source
Extracted wood	*dext*	0.736	3 main commercial species	[31, 32]^a^
	$f_{SW}$	0.33	No uncertainty reported	[33]^b^
Sawnwood decay	$\lambda _{SW}$	$N_{t0}(30,15^2)$	Positive normal distribution	[30]^c^
Damage	$\theta _0$	$N(1.28, 0.48^2)$	Normal approximation of the posterior	TmFO sites^b^
	$\theta _1$	$N(0.56,0.14^2)$	Normal approximation of the posterior	TmFO sites^b^
	$\sigma _{dam}$	$N(0.37,0.04^2)$	Normal approximation of the posterior	TmFO sites^b^
Logging roads	$\delta$	*U*(0.12, 0.25)	Personal communication	NFS expertise^a^
	$\eta$	*exp*(17.95)	Decreasing monotonic distribution	[36]^a^
Main skid trails	*SurfST*	*U*(0.04, 0.10)	Only range of values is reported	[36]^a^
	$f_{50}$	*Beta*(15.10, 15.32)	Proportion	Guyafor^a^
Logging decks	*LDeck*	$ACS\times lnN(-6.12,0.58)$	Positive distribution	Original data^a^
LWN decay	*fLWN*	*Beta*(1115, 169)	Proportion	Guyafor^a^
	$\lambda _1$	$N(0.069,0.028^2)$	Normal approximation of the posterior	Guyafor^a^
	$\lambda _2$	$N(0.198,0.074^2)$	Normal approximation of the posterior	Guyafor^a^
	$\pi _1$	$N(0.512,0.032^2)$	Normal approximation of the posterior	Guyafor^a^
	$\sigma _{LWN}$	$N(0.015,0.0035^2)$	Normal approximation of the posterior	Guyafor^a^
FWN decay	$\lambda _{FWN}$	$N(0.19, 0.026^2)$	Reported distribution	[37]^c^
Forest recovery	$\alpha$	$N(1.100, 0.03^2)$	Reported distribution	[13]^c^
Regeneration	$\mu _0$	$N(-4.505,0.124^2)$	Maximum likelihood estimate	Arbocel^a^
	$\mu _1$	$N(1.085,0.038^2)$	Maximum likelihood estimate	Arbocel^a^
	$\varphi$	490.7	Maximum likelihood estimate	Arbocel^a^

Parameters are grouped by submodel in which they appear. *FWN* fine woody necromass decay;* LWN* large woody necromass. *ACS* initial above-ground carbon stock (MgC·ha$^{-1}$). Parameters are: *dext* mean density of extracted roundwood in French Guiana; $f_{SW}$ efficiency of wood transformation in sawmills; $\lambda _{SW}$ decay rate of sawnwood; $(\theta _0, \theta _1, \sigma _{dam})$ parameters of the relationship between extracted wood and logging damage; $(\eta ,\delta )$ length (hm·ha$^{-1}$) and width (hm) of logging roads; *SurfST* main skid trails area (ha); $f_{50}$ proportion of above-ground carbon in trees DBH <50 cm; *LDeck* carbon loss in logging decks; $f_{LWN}$ fraction of necromass carbon in large woody necromass; $(\lambda _1,\lambda _2, \pi _1, \sigma _{LWN)})$ parameters of large woody necromass decay; $\alpha$ parameter of the recovery model; $\mu _0$, $\mu _1$ and $\varphi$: parameters of the regeneration beta model

^a^ Valid in French Guiana

^b^ Valid in Amazonia

^c^ Valid in the tropics

*Logging decks* Logging decks are cleared to store harvested boles. Based on detailed mapping of 11 logging decks in logging units HKO95 (283 ha) and HKO96 (466 ha) and with the information that one logging deck is set up for every 70 ha (NFS expertise, personal communication), we used:

14 $$\begin{aligned} \dfrac{LDeck_{p}}{ACS_p} \sim lnN(-6.12, 0.58^2) \end{aligned}$$

with $ACS_p$ the above ground carbon stock of the logging unit *p*, and $LDeck_p$ (MgC·ha$^{-1}$) is the carbon removed in logging decks.

#### Dead wood decay

Assuming that all woody debris are left out for decay in situ, dead wood was divided into two pools with different decay rates: (i) fine woody necromass (FWN), i.e. leaves, twigs, and branches with diameter <10 cm, and (ii) large woody necromass (LWN), i.e. logs and branches with diameter ≥10 cm.

*Fraction of Large Woody Necromass* We used Chambers et al. [37] model to estimate the fraction of large woody necromass $LWN_i$ at tree scale (*i*).

For a tree *i* : If $DBH_i<100$ cm:

15 $$\begin{aligned} LWN_i = 0.774+0.0018\times DBH_i \end{aligned}$$

else $$LWN_i=0.95$$.

This fraction of large woody necromass has been estimated for all individuals in control plots of the 11 Guyafor sites. For one site *j*, the fraction of large woody necromass is:

16 $$\begin{aligned} fLWN_{j} = \dfrac{\sum \nolimits _i (LWN_{i,j}\times ACS_{i,j})}{\sum \nolimits _i ACS_{i,j}} \end{aligned}$$

We report the obtained distribution of $fLWN_j$ in Table 1.

At $t=t0_p$ ($t0_p$: year of logging) in the logging unit *p*:

17 $$\begin{aligned} FWN_{p,t0_p} = (1-f_{LWN})\times (Dam_p+Road_p+STrail_p+LDeck_p) \end{aligned}$$

18 $$\begin{aligned} LWN_{p,t0_p} = f_{LWN}\times (Dam_p+Road_p+STrail_p+Ldeck_p) \end{aligned}$$

where $FWN_{p,t0_p}$ and $LWN_{p,t0_p}$ (MgC·ha$^{-1}$) are the carbon in fine and large woody necromass, respectively, left on the logging unit *p* at $t0_p$.

*Decay of Fine Woody Necromass* Fine woody necromass decay in logging unit *p* at time *t* is estimated by the following model [38]:

19 $$\begin{aligned} DecFWN_{p,t}=FWN_{p,t0_p}\times exp \Big ( -\lambda _{FWN}\times (t-t\textit{0}) \Big ) \end{aligned}$$

where $DecFWN_{p,t}$ (MgC·ha$^{-1}$) is the fine woody necromass left in the logging unit *p* at time *t* and $\lambda _{FWN}$ is the decay rate of fine woody necromass (Table 1).

*Decay of Large Woody Necromass* There was no published model for decay of large woody necromass at the logging unit level and derived individual tree large woody necromass (LWN) decay from [4]:

20 $$\begin{aligned} LWN_{i,t} = LWN_{i,t0}\times exp \Big (-\frac{\gamma _1}{WD_i^{\gamma _2} \times (circ_i)^{\gamma _3}}\times (t-t0) \Big ) \end{aligned}$$

where $LWN_{i,t}$ is the large woody necromass of the individual *i* at time *t*, $WD_i$ the wood density of the individual *i* and $circ_i$ the circumference at breast height of the individual *i*, and $(\gamma _k)_{1,2,3}$ are parameters of the model [4]. For all 11 Guyafor sites, the model was applied to all individuals *i* of the control plots. Individual trajectories are then aggregated to have the large woody necromass left at time *t* on the site *j*:

21 $$\begin{aligned} LWN_{j,t}= \sum \limits _i LWN_{i,j,t} \end{aligned}$$

where $LWN_{j,t}$ is the large woody necromass left at time *t* on the site *j*; $LWN_{i,j,t}$ is the large woody necromass at time *t* of the individual *i* from the site *j*.

A new model was developed at the logging unit level: to better fit the data, a double-decay model was chosen instead of a one-parameter decay model, because it drastically reduced the bias in our predictions. The double-decay model works as if there were two pools of large woody necromass (i.e. sap- and heart-wood) with two different decay rates.

22 $$\begin{aligned} \frac{DecLWN_{p,t}}{LWN_{p,t0}} = \pi _1 \times exp \Big (- \lambda _1 \times (t-t0_p) \Big ) + (1 - \pi _1) \times exp \Big (- \lambda _2 \times (t-t0_p) \Big ) + \varepsilon _{LWN,\,p} \end{aligned}$$

with

$$\begin{aligned} \pi _1>0.5 \end{aligned}$$

and

$$\begin{aligned} \varepsilon _{LWN,p} \sim N(0;\sigma _{LWN}) \end{aligned}$$

where $\dfrac{DecLWN_{p,t}}{LWN_{p,t0_p}}$ is the fraction of the initial large woody necromass left on logging unit *p* at time *t* ; $\lambda _1$ and $\lambda _2$ are the two decay rates and $\pi _1$ the fraction of large woody necromass decomposing with the rate $\lambda _1$; $\varepsilon _{LWN,p}$ is the additive error for the logging unit *p*, taken in $N(0;\sigma _{LWN}^2)$, the centred normal distribution of standard deviation $\sigma _{LWN}$.

Parameters $(\lambda _1; \lambda _2; \pi _1;\sigma _{LWN} )$ were estimated as follows: (i) 100 sets of parameters $(\gamma _k)_{1,2,3}$ of the individual decay model were taken in their distribution; (ii) for each set of individual parameters $(\gamma _k)_{1,2,3}$, the trajectories of large woody necromass $LWN_{j,t}$ for each site *j* in the 11 Guyafor sites was estimated; (iii) parameters $(\lambda _1; \lambda _2; \pi _1;\sigma _{LWN})$ were then estimated using a Bayesian inference with a Markov Chain Monte Carlo method. Uninformative priors were chosen. One set of parameters $(\lambda _1; \lambda _2; \pi _1;\sigma _{LWN})$ was stored for every set of individual parameters $(\gamma _k)_{1,2,3}$. 100 sets of parameters $(\lambda _1; \lambda _2; \pi _1;\sigma _{LWN})$ were then stored to be used in the full model (the normal approximations of the posterior distributions are in Table 1).

### Live biomass

#### Carbon recovery in logged areas

The assumption of a linear carbon recovery was made, i.e. the rate at which a logged area stores above-ground carbon after logging is constant and becomes null at recovery time, when the above-ground carbon has reached its original level. This assumption is arguable but is consistent with available data on post-logging dynamics in the region [13]. Under this assumption, the recovery time (*RT*) can be estimated with the sole percentage of above-ground carbon loss caused by logging:

23 $$\begin{aligned} RT_p = \left( \frac{100 \times (Ext_p+Dam_p)}{ACS_p}\right) ^{\alpha } \end{aligned}$$

with

24 $$\begin{aligned} \alpha \sim \mathcal {N} (1.100, 0.013^2) \end{aligned}$$

with $RT_p$ (yr) the recovery time of the logging unit *p*, $Ext_p+Dam_p$ (MgC·ha$^{-1}$) the above-ground carbon loss per ha logged in the logging unit *p* and $ACS_p$ the initial above-ground carbon stock (MgC·ha$^{-1}$).

The above-ground carbon recovered $Recov_{p,t}$ (MgC·ha$^{-1}$) at time *t* in the logged area of the logging unit *p* is then :

25 $$\begin{aligned} Recov_{p,t} = \min \left( Ext_p+Dam_p \text {;} (t-t0_p) \times \frac{Ext_p+Dam_p}{RT_p}\right) \end{aligned}$$

When $t-t0_p \le RT_p$ (i.e. before recovery time), the logged area stores carbon with a rate $\dfrac{Ext_p+Dam_p}{RT_p}$. When $t-t0_p > RT_p$, the logged area has recovered all the above-ground carbon lost $Ext_p+Dam_p$ and the carbon storage stops.

#### Regeneration on abandoned main skid trails and logging decks

**Fig. 5:**
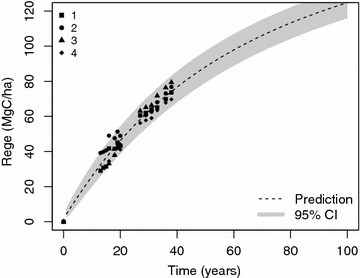
Beta model of carbon regeneration (*Rege*, MgC·ha$^{-1}$) on four clearcut plots. The* dashed line* is the prediction with maximum likelihood, the shaded area is the 95 % credibility interval on the prediction. Data are taken from four 1.65-ha plots in Arbocel site (French Guiana), clearcut 38 year ago and let to natural regeneration

The fate of the carbon dynamics on abandonned skid trails and logging decks was modelled taking advantage of four 1.65-ha plots clearcut 38 years ago (Fig. 5), and regularly measured since [28]. Carbon stocks regeneration on skid trails with compacted soils may be expected to be slower than on large clear-cuts but no data on skid trails reforestation have been produced in French Guiana. The following model was inferred:

26 $$\begin{aligned} Rege_{p,t} = ACS_p \times Y_{p,t} \end{aligned}$$

with

27 $$\begin{aligned} Y_{p,t} \sim Beta \Big (\mu _0+\mu _1 \times log(t-t0_p+1)\text {;} \varphi \Big ) \end{aligned}$$

where $Rege_{p,t}$ (MgC·ha$^{-1}$) is the above-ground carbon gain from forest regeneration on plot *p* at time *t*, $ACS_p$ (MgC·ha$^{-1}$) is the initial above-ground carbon stock of the plot *p* (i.e. before the clear cut), $Y_{p,t}$ is the proportion of the initial above-ground carbon stock recovered on the plot *p* at time *t*. $Y_{p,t}$ follows a beta distribution of mean $\mu _0+\mu _1 \times log(t-t0_p+1)$ and of precision $\varphi$ (parameters ($\mu _0$, $\mu _1$, $\varphi$) estimated distributions are in Table 1). The beta distribution is defined on [0, 1] and takes two shape parameters.

### Accounting for uncertainties

#### Uncertainty propagation

The model was built and parametrised with MCMC algorithms in order to track uncertainties. Uncertainty propagation was done with the following steps: (i) every parameter is randomly taken in its distribution; (ii) the model is applied over all logging units of the region with these parameters values; (iii) results are integrated in space and time and stored. These three steps are repeated 5000 times and summary statistics are then calculated.

#### Sensitivity analysis

To assess the uncertainty induced by each submodel, we carried out a sensitivity analysis where the parameters of each submodel are set to their maximum likelihood values one after the other, making the submodel deterministic while the rest of the model remains stochastic. The results for each deterministic submodel are then compared to the total model: the “shrinkage” of their summary statistics reflects the uncertainty linked to the parameters of a specific submodel, more than the model and modelling choices themselves.

## Results and discussion

### Methodological aspects

In this study we developed a new methodology to assess the above-ground carbon balance of tropical managed forests. The model is based on a book-keeping approach: it aggregates post-logging carbon fluxes of every logging unit over a given area and period of time to obtain the regional carbon balance. The main advantage of our methodology compared to other published methods estimating emissions from selectively logged concessions [24, 25] lies in the fact that both post-logging carbon sequestration and necromass respiration are explicit functions of time. This framework is flexible enough to be adapted to new situations and areas, but potential users should keep in mind that applying this model to other areas will require in-depth analysis on the suitability of the parameters value, variables distribution and submodels choices. The framework can also be adapted to integrate new information on post-logging forest dynamics or to take into account new logging technique, e.g. new techniques of reduced impact logging (RIL) likely to decrease the quantity of damage for the same logging intensity [39] but used in less than 5 % of selectively logged areas so far [1]. Part of logging damage may also be used for new purposes, e.g. burned in energy plants as wood fuel [40]. The model can be adapted and re-parametrised very quickly to take into account such changes.

We aknowledge that French Guiana is a special case in the tropics in regards to data quality and availability. It is therefore easier to develop a methodology there than in other countries where both input and calibration data are scarse. Submodels were either taken from the literature or specifically developed for this study with data at regional and continental scales. For each submodel, parameters are considered as uncertain quantities making good use of the Bayesian inference framework. Depending on spatial extent of the data used to calibrate the corresponding submodel, parameters distribution have different geographic validity (Table 1). Submodels from the literature and our submodel of logging damage (*Dam*) are considered to be valid over Amazonia. The estimated distribution of corresponding parameters could therefore be used for assessing the carbon balance of any Amazonian region. The other models developed in this study [deforestation on roads, main skid trails and decks, large woody necromass (LWN) decay and forest regeneration] were calibrated with data from a regional plot network, Guyafor or with NFS data and should be used with caution elsewhere. In order to study the carbon balance of other regions, data from permanent plots in the region of interest should be used to validate, and if necessary re-calibrate submodels.

The method is simple and only requires two sets of input data : logged areas in space and time and the intensities at which these forest areas were logged. These data can come either from official logging statistics or from remote sensing methods. In many countries, logging operations occur almost exclusively inside logging concessions for which management plans do exist. For instance, in the Congo Basin, 45 Mha of forest are managed under the concession system [41]. In these cases, the logging statistics needed can be taken directly from management plans. In other countries where management plans do not exist or are not fully applied [42], remote sensing tools can be useful to map logged areas. For instance, Asner et al. [5] tracked logged areas in five States of the Brazilian Amazon during three consecutive years using Landsat imagery and CLAS methodology. Logging intensities are more difficult to obtain with remote sensing methods: a way to overcome this problem is to use region-wide statistics on commercialized wood that are generally quite robust because wood boles from either legal or illegal logging eventually need authorizations to be transported and commercialized. The simplest way to estimate logging intensities is then to use an average logging intensity, or to use local expertise to locally refine this average value. Bayesian approaches are quite appropriate to mix *a priori* knowledge with quantitative datasets. For both logged areas and and logging intensities, a close attention should be paid to uncertainties and their propagation into the complete model.

### Carbon balance at the logging unit scale

**Fig. 6:**
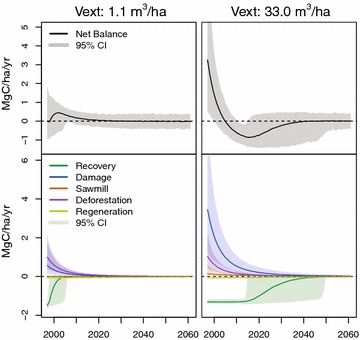
Annual above-ground carbon fluxes from logging on a 65-year period in two logging units logged in 1994 with different logging intensities. The first logging unit underwent a low-intensity logging (*left*) with a mean extracted volume $V_{ext}=1.1$ m$^3$·ha$^{-1}$whereas the second logging unit underwent a high-intensity logging (*right*) with a mean extracted volume $V_{ext}=33.0$ m$^3$·ha$^{-1}$. For each plot, the net carbon flux (*top*) is the result of four components: emissions from logging damage (*blue*), emissions from road, deforestation on main skid trail and logging deck (*purple*), emissions from extracted timber (*red*), accumulation from post-logging recovery on the plot (*forest green*) and accumulation from regeneration on main skid trails and logging decks (*light green*). The* shaded areas* correspond to the 95 % credibility intervals

To fully understand the theoretical behaviour of our model we focused on two logging units both logged in 1996: one with a low logging intensity (1.1 m$^3$·ha$^{-1}$) and one with a high logging intensity (33 m$^3$·ha$^{-1}$). For those two logging units, we estimated carbon fluxes (MgC·ha$^{-1}$·year$^{-1}$) during an 65-year period, which corresponds to the official cutting cycle in French Guiana (Fig. 6).

#### Annual fluxes

At low logging intensity, the two main sources of carbon are both logging damage and deforestation (on roads and main skid trails). At higher logging intensities, emissions from deforestation become negligible compared to emissions from damage, consistent with previous remote-sensing results in the Brazilian Amazon [43]. According to our model, carbon fluxes reach a steady state before the end of the 65-year period for both logging units (Fig. 6).

The different trends of the net carbon change are mostly explained by the interplay of two components: emissions from logging damage and accumulation from recovery in logging gaps. On one hand, higher logging intensities obviously cause more logging damage, resulting in higher carbon emissions from damage decay. On the other hand, the annual carbon flux from recovery in logging gaps is less dependent on logging damage (Fig. 6): a logging unit that has undergone high logging intensities will thus take longer to recover.

Even under high logging intensity (33 m$^3$·ha$^{-1}$), the time to null carbon fluxes does not exceed 50 year. Previous results from satellite data have shown that in the Brazilian Amazon, where logging intensities usually range between 20 and 30 m$^3$·ha$^{-1}$, carbon emissions last for 2–3 decades [14]. This last study also showed that carbon forest recovery may last for up to a century, twice as much as our highest prediction (Fig. 6). This difference in recovery times may arise from the model of forest recovery chosen in our study. We chose the model proposed in Rutishauser et al. [13], that assumes a constant recovery rate. With lack of insight in long-term dynamics of recovering forests, we made the conservative assumption that the carbon accumulation stops when initial carbon stock is recovered. However it is possible that recovering forests behave differently when the carbon stock approaches its initial value. The carbon storage could have a logistic behaviour, decelerating when approaching the initial carbon stock, or could oscillate around an equilibrium value (i.e. the initial carbon stock) and stabilize only after a longer time period [44].

Under the assumptions of our model, the carbon fluxes of a logged forest return to their equilibrium state before the end of the cutting cycle. At second harvest cycle, the carbon stocks will likely be close to their initial value, except on logging roads and skid trails. This does not mean that the forest will have returned to its original state as selective logging is known to have long-lasting effects on forest structure, timber volume and biodiversity [45].

#### Cumulative fluxes

**Fig. 7:**
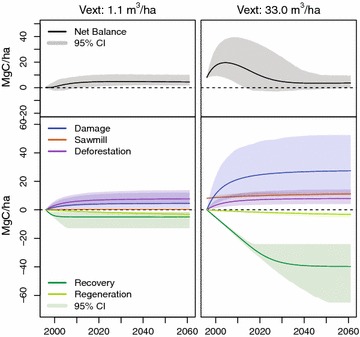
Above-ground carbon balance from logging on a 65-year period in two logging units logged in 1994 with different logging intensities. The carbon balance at time *t* is the integral of annual carbon fluxes, i.e. the sum of annual carbon fluxes from the year of logging (1996) to *t*. The first logging unit underwent a low-intensity logging (*left*) with a mean extracted volume $V_{ext}$=1.1 m$^3$·ha$^{-1}$ whereas the second logging unit underwent a high-intensity logging (*right*) with a mean extracted volume $V_{ext}=33.0$ m$^3$·ha$^{-1}$. For each plot, the net carbon balance (*top*) is the result of fourcomponents: emissions from logging damage (*blue*), emissions from road, deforestation on main skid trail and logging deck (*purple*), emissions from extracted timber (*red*), accumulation from post-logging recovery on the plot (*forest green*) and accumulation from regeneration on main skid trails and logging decks (*light green*). The* shaded areas* correspond to the 95 % credibility intervals

To assess the carbon balance of our two logging units, we estimated the cumulative carbon fluxes (Fig. 7). The cumulative carbon flux at time *t* is the sum of annual carbon fluxes between the logging year and *t*.

The carbon balance of the first logging unit (with low logging intensity) is not different from zero in the first years because the recovery rate is quite similar to emissions from dead wood decay and deforestation. At the end of the cutting cycle, the carbon balance of the logging unit is about equal to the carbon emissions from deforestation (on roads and skid trails). The second logging unit (with high logging intensity) has high emissions from damage decay in the first 8 years after logging, that exceed carbon accumulation from forest recovery: the carbon balance of the logging unit increases. Between 8 and 40 years after logging, carbon accumulation from forest recovery exceeds carbon emissions: the carbon balance decreases. After 40 years, the carbon balance of the logging unit stabilizes at a value about equal to the emissions from deforestation.

In the first years after logging, the net carbon balance is mostly driven by the logging damage, directly related to logging intensity. High logging intensities cause more damage, and thus induce higher emissions from dead wood decay. At the end of the cutting cycle, both logging units have the same carbon balance, corresponding to the carbon emissions from deforestation on logging roads and main skid trails. Indeed, there is little regeneration in main skid trails, and no regeneration in deforested roads whereas carbon recovery in logging gaps compensates the carbon emissions from extracted wood and logging damage. In terms of forest management, the carbon balance of a logging unit at the end of a cutting cycle thus depends mostly on the total surface of logging roads and skid trails. To mitigate carbon emissions, logging should be planned to avoid road and skid trail opening. This can be done by intensifying timber extraction per logged area and thus lowering the amount of infrastructure needed to get the logs out of the area.

### Carbon balance at the regional scale

**Fig. 8:**
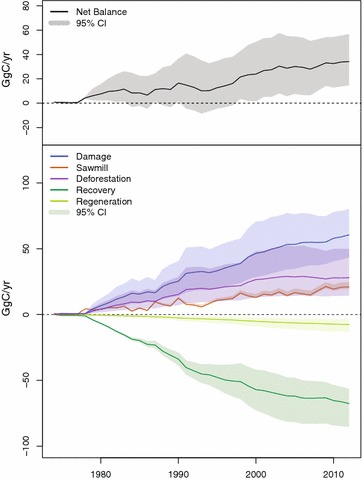
Annual above-ground carbon fluxes from logging in the Permanent Forest Estate between 1974–2012. The net carbon flux (*top*) is the result of four components: emissions from logging damage (*blue*), emissions from road, deforestation on main skid trail and logging deck (*purple*), emissions from extracted timber (*red*), accumulation from post-logging recovery on the plot (*forest green*) and accumulation from regeneration on main skid trails and logging decks (*light green*). The* shaded areas* correspond to the 95 % credibility intervals

**Fig. 9:**
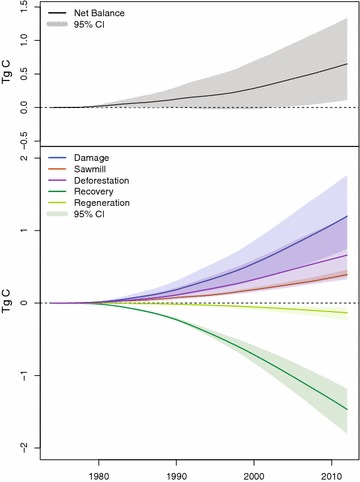
Above-ground carbon balance of the French Guiana Permanent Forest Estate between 1974–2012. The carbon balance at time *t* is the integral of annual carbon fluxes, i.e. the sum of annual carbon fluxes from the first year (1974) to *t*. The net carbon balance (*top*) is the result of four components: emissions from logging damage (*blue*), emissions from road, deforestation on main skid trail and logging deck (*purple*), emissions from extracted timber (*red*), accumulation from post-logging recovery on the plot* (forest gree*n) and accumulation from regeneration on main skid trails and logging decks (*light green*). The* shaded areas* correspond to the 95 % credibility intervals

After assessing carbon fluxes for each logged logging unit, results were aggregated over space to assess the carbon fluxes over the whole Permanent Forest Estate, and over time to assess the carbon balance between 1974 and 2012 (Figs. 8, 9).

#### Annual fluxes

As for the logging unit level, the largest carbon emissions arise from the decay of logging damage (Fig. 8): this is consistent with results from field data on other Amazonian countries [25]. Carbon accumulation is mainly explained by forest recovery in the logged area, forest regeneration on main skid trails and logging decks being negligible.

Net carbon fluxes are positive: the Permanent Forest Estate in French Guiana behaves like a carbon source during the 1974–2012 period. However zero is included in the 95 % credibility interval until 2000: the positiveness of carbon fluxes is not significant between 1974 and 2000. In the mid 1990’ there is a sudden increase in net carbon fluxes, and after 2000 the lower bound of the 95 % credibility interval is positive: between 2000 and 2012, logging in the Permanent Forest Estate significantly emitted carbon to the atmosphere. This change in the trend of net carbon flux corresponds to increased logging intensities in the mid 1990s (Fig. 2).

#### The carbon balance at the regional scale

Selective logging countrywide has induced significant carbon emissions since 2000 (Fig. 8), resulting in a positive net carbon balance in 2012 (Fig. 9). The net above-ground carbon balance in 2012 is estimated to be comprised between 0.12 and 1.33 Tg C, with a median value of 0.64 Tg C. With 1.36 Pg C stored in above-ground carbon [29], selective logging has emitted between 0.01 and 0.10 % of the French Guiana forest stock over the 1974–2012 period. Such ratio may look quite low when compared to the above-ground carbon stock of all the total forested area, but we have to keep in mind that the units logged over the 1974–2012 in the Permanent Forest Estate cover 0.12 Mha, i.e. 5 % of the total forested area in French Guiana (Fig. 1). Moreover, logging intensities in French Guiana are low (12.7 m$^3$·ha$^{-1}$·year$^{-1}$, Fig. 2) compared to the Brazilian Amazon where logging intensities average 23.2 m$^3$·ha$^{-1}$·year$^{-1}$ [5]. Applying our model to the rest of Amazonian managed forests should thus raise higher carbon emissions.

### Possible model improvements

#### Deforestation, damage and LWN decay are key submodels

**Fig. 10:**
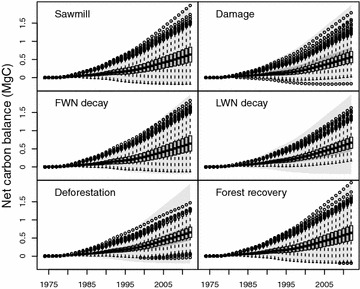
Estimating the uncertainty due to each submodel into the net carbon balance. The box plots represent the distribution of the net carbon balance when parameters of each submodel are set to their maximum likelihood value. The shaded area is the range of the net carbon balance for the total model, i.e. when all parameters are taken in their statistical distribution

The sensitivity analysis reveals three main sources of uncertainty in our model: deforestation, large woody necromass decay and logging damage (Fig. 10). Despite relatively low values of logging areas that are annually deforested, the key role of the deforestation submodel in generating the net carbon balance uncertainties (Fig. 10) may look quite surprising. Because the carbon loss on permanently-deforested logging roads will never be recovered, carbon emissions accumulate over time, and their uncertainties as well. At the end of the 40-year period, the cumulative uncertainties then become non-negligible. The quantity of damage intervenes in two carbon fluxes: emissions from logging damage decay and carbon recovery (Fig. 3). If the objective is to estimate one of these two fluxes, efforts should be made to improve the prediction of logging damage. Until now the only explanatory variable of our logging damage model is the logging intensity *Vext* (m$^3$·ha$^{-1}$). A great proportion of the variability remains unexplained, and other factors may help improve the prediction of logging damage. For instance, including further information such as the logging techniques applied (conventional or reduced-impact logging), the local topography, or the forest structure would certainly have improved damage predictions. Nevertheless, the main objective is to estimate the net carbon balance, improving the estimation of logging damage should not be a priority. Indeed, recovery dynamics are very fast, lasting for a few decades at the most (Fig. 6). At the regional scale, emissions from damage decay are almost instantaneously mitigated by recovery mechanisms (Figs. 8, 9), so the variability of logging damage has little influence on the overall variability of the carbon balance (Fig. 10). On the contrary, the variability of large woody necromass decay explains a great part of the overall uncertainty of the total carbon balance (Fig. 10). In this submodel, uncertainties come from the individual-based model [4] and from the error propagation during the passage to the logging unit scale. To reduce uncertainties over the large woody necromass decay model, direct field measurements should be made to estimate the decay rate directly at the logging unit scale.

#### Below-ground carbon

This study remains restricted to aboveground carbon, the main carbon pool in tropical forests [46], but below-ground carbon (roots, soil) could also be impacted by selective logging and result in carbon emissions. Some studies suggest that soil carbon stocks may decrease after selective logging [47], but carbon sequestration in roots and in the woody debris that are not respired and that return to soil could also be expected [48]. In this study we do not consider below-ground carbon fluxes due to lack of robust information. Gathering more information and adding below-ground carbon dynamics into the model would further improve the model.

## Conclusions

We here provide a comprehensive methodology to estimate above-ground carbon fluxes in managed tropical forests at both local and regional scales, easily replicable in other tropical regions. This is a further contribution towards more accurate assessment of the carbon budget of tropical managed forests. The framework is also flexible and easy to adapt to new models: in particular, new logging techniques or new management practices (*e.g. fuel wood*) could be added to the model. This can be useful when investigating the carbon balance of upcoming opportunities and of future management choices for tropical production forests.

## References

[1] Putz FE, Zuidema PA, Pinard MA, Boot RG, Sayer JA, Sheil D (2008). Improved tropical forest management for carbon retention. PLoS Biol.

[2] Blaser J, Sarre A, Poore D, Johnson S. Status of tropical forest management 2011, vol 38. ITTO; 2011. p. 27–31.

[3] Mertz O, Müller D, Sikor T, Hett C, Heinimann A, Castella JC (2012). The forgotten D: challenges of addressing forest degradation in complex mosaic landscapes under REDD+. Geografisk Tidsskrift-Danish J Geogr.

[4] Hérault B, Beauchêne J, Muller F, Wagner F, Baraloto C, Blanc L (2010). Modeling decay rates of dead wood in a neotropical forest. Oecologia.

[5] Asner GP, Knapp DE, Broadbent EN, Oliveira PJC, Keller M, Silva JN (2005). Selective logging in the Brazilian Amazon. Science.

[6] Picard N, Gourlet-Fleury S, Forni É (2012). Estimating damage from selective logging and implications for tropical forest management. Can J For Res.

[7] Sist P, Ferreira FN (2007). Sustainability of reduced-impact logging in the Eastern Amazon. For Ecol Manag.

[8] Sist P, Mazzei L, Blanc L, Rutishauser E (2014). Large trees as key elements of carbon storage and dynamics after selective logging in the Eastern Amazon. For Ecol Manag.

[9] Shenkin A, Bolker B, Peña-Claros M, Licona JC, Putz FE (2016). Fates of trees damaged by logging in Amazonian Bolivia. For Ecol Manag.

[10] Rutishauser E, Hérault B, Petronelli P, Sist P. Tree height reduction after selective logging in a tropical forest. Biotropica. 2016;48(3):285–9.

[11] Hérault B, Ouallet J, Blanc L, Wagner F, Baraloto C (2010). Growth responses of neotropical trees to logging gaps. J Appl Ecol.

[12] Blanc L, Echard M, Herault B, Bonal D, Marcon E, Chave J (2009). Dynamics of aboveground carbon stocks in a selectively logged tropical forest. Ecol Appl.

[13] Rutishauser E, Hérault B, Baraloto C, Blanc L, Descroix L, Sotta ED (2015). Rapid tree carbon stock recovery in managed Amazonian forests. Curr Biol.

[14] Huang M, Asner GP. Long-term carbon loss and recovery following selective logging in Amazon forests. Glob Biogeochem Cycles. 2010;24(3):1–15.

[15] Kleinschroth F, Gourlet-Fleury S, Sist P, Mortier F, Healey JR (2015). Legacy of logging roads in the Congo Basin: how persistent are the scars in forest cover?. Ecosphere.

[16] Goetz SJ, Baccini A, Laporte NT, Johns T, Walker W, Kellndorfer J (2009). Mapping and monitoring carbon stocks with satellite observations: a comparison of methods. Carbon Balance Manag.

[17] Asner GP, Keller M, Pereira R, Zweede JC (2002). Remote sensing of selective logging in Amazonia. Remote Sens Environ.

[18] Asner GP, Powell GVN, Mascaro J, Knapp DE, Clark JK, Jacobson J (2010). High-resolution forest carbon stocks and emissions in the Amazon. Proc Natl Acad Sci.

[19] Réjou-Méchain M, Muller-Landau HC, Detto M, Thomas SC, Le Toan T, Saatchi SS (2014). Local spatial structure of forest biomass and its consequences for remote sensing of carbon stocks. Biogeosci Discuss.

[20] Peres CA, Barlow J, Laurance WF (2006). Detecting anthropogenic disturbance in tropical forests. Trends Ecol Evol.

[21] Pereira R, Zweede J, Asner GP, Keller M (2002). Forest canopy damage and recovery in reduced-impact and conventional selective logging in eastern Para, Brazil. For Ecol Manag.

[22] Meyer V, Saatchi SS, Chave J, Dalling JW, Bohlman S, Ga Fricker (2013). Detecting tropical forest biomass dynamics from repeated airborne lidar measurements. Biogeosciences.

[23] IPCC. 2006 IPCC guidelines for national greenhouse gas inventories, chapter 4. 2006, p. 4.14.

[24] Griscom B, Ellis P, Putz FE (2014). Carbon emissions performance of commercial logging in East Kalimantan, Indonesia. Glob Change Biol.

[25] Pearson TRH, Brown S, Casarim FM (2014). Carbon emissions from tropical forest degradation caused by logging. Environ Res Lett.

[26] Khun V, Sasaki N (2014). Cumulative carbon fluxes due to selective logging in Southeast Asia. Low Carbon Econ.

[27] Sist P, Rutishauser E, Peña-Claros M, Shenkin A, Hérault B, Blanc L (2014). The tropical managed forests observatory: a research network addressing the future of tropical logged forests. Appl Veg Sci.

[28] Toriola D, Chareyre P, Buttler A (1998). Distribution of primary forest plant species in a 19-year old secondary forest in French Guiana. J Trop Ecol.

[29] Guitet S, Hérault B, Molto Q, Brunaux O, Couteron P (2015). Spatial structure of above-ground biomass limits accuracy of carbon mapping in rainforest but large scale forest inventories can help to overcome. PLoS One.

[30] Penman J, Gytarsky M, Hiraishi T, Krug T. Good practice guidance for land use, land-use change and forestry. Intergovernmental Panel on Climate Change (IPCC), chapter 3, Appendix 3.A.1.3. UNEP; 2003. p. 268–70.

[31] Ollivier M, Baraloto C, Marcon E (2007). A trait database for Guianan rain forest trees permits intra- and inter-specific contrasts. Ann For Sci.

[32] Fargeon H, Aubry-kientz M, Brunaux O, Descroix L, Guitet S, Rossi V (2016). Vulnerability of commercial tree species to water stress in logged forests of the Guiana shield. Forests.

[33] Keller M, Asner GP, Silva JNM, Palace M. Sustainability of selective logging of upland forests in the Brazilian Amazon: carbon budgets and remote sensing as tools for evaluation of logging effects. In: Working forests in the tropics: conservation through sustainable management? New York: Columbia University Press; 2004. p. 41–63.

[34] Asner GP, Keller M, Lentini M, Merry F, Souza CJ (2009). Selective logging and its relation to deforestation. Geophys Monogr Ser.

[35] Eshun JF, Potting J, Leemans R (2011). LCA of the timber sector in Ghana: preliminary life cycle impact assessment (LCIA). Int J Life Cycle Assess.

[36] Guitet S, Pithon S, Brunaux O, Jubelin G, Gond V (2012). Impacts of logging on the canopy and the consequences for forest management in French Guiana. For Ecol Manag.

[37] Chambers JQ, Higuchi N, Teixeira LM, dos Santos J, Laurance SG, Trumbore SE (2004). Response of tree biomass and wood litter to disturbance in a Central Amazon forest. Oecologia.

[38] Chambers JQ, Higuchi N, Schimel JP, Ferreira LV, Melack JM (2000). Decomposition and carbon cycling of dead trees in tropical forests of the central Amazon. Oecologia.

[39] Putz FE, Sist P, Fredericksen T, Dykstra D (2008). Reduced-impact logging: challenges and opportunities. For Ecol Manag.

[40] Berndes G, Hoogwijk M, van den Broek R (2003). The contribution of biomass in the future global energy supply: a review of 17 studies. Biomass Bioenerg.

[41] Karsenty A, Vermuelen C (2016). Toward “Concessions 2.0”: articulating inclusive and exclusive management in production forests in Central Africa. Int For Rev.

[42] Monteiro A, Cardoso D, Conrado D, Veríssimo A, Souza C. Forest management transparency report—State of Pará (2012–2013). Imazon; 2013. http://imazon.org.br/publicacoes/forest-management-transparency-reportstate-of-para-2011-to-2012/?lang=en.

[43] Asner GP, Keller M, Silva J (2004). Spatial and temporal dynamics of forest canopy gaps following selective logging in the eastern Amazon. Glob Change Biol.

[44] Holling CS (1973). Resilience and stability of ecological systems. Annu Rev Ecol Syst.

[45] Putz FE, Pa Zuidema, Synnott T, Peña-Claros M, Ma Pinard, Sheil D (2012). Sustaining conservation values in selectively logged tropical forests: the attained and the attainable. Conserv Lett.

[46] Pan Y, Birdsey RA, Phillips OL, Jackson RB (2013). The structure, distribution, and biomass of the World’s forests. Annu Rev Ecol Evol Syst.

[47] Chiti T, Perugini L, Vespertino D, Valentini R (2015). Effect of selective logging on soil organic carbon dynamics in tropical forests in central and western Africa. Plant Soil.

[48] Chambers JQ, Schimel JP, Nobre CA (2001). Respiration from coarse wood litter in central Amazon forests. Biogeochemistry.

